# Pilomatricoma of the elbow

**DOI:** 10.1093/jscr/rjaf457

**Published:** 2025-07-01

**Authors:** Youssef El Hassnaoui, Abdelaziz E L Ansari, Mohamed Tazi, Ahmed El Ghazzal, Hamza Madani, Issam Boulazaib, Hicham Ait Benali, Mohammed Shimi

**Affiliations:** Orthopedics and Trauma Surgery Department, Mohammed VI University Hospital Center Tangier, Faculty of Medicine and Pharmacy, Abdelmalek Essaadi University, Tangier, 90000, Morocco; Orthopedics and Trauma Surgery Department, Mohammed VI University Hospital Center Tangier, Faculty of Medicine and Pharmacy, Abdelmalek Essaadi University, Tangier, 90000, Morocco; Orthopedics and Trauma Surgery Department, Mohammed VI University Hospital Center Tangier, Faculty of Medicine and Pharmacy, Abdelmalek Essaadi University, Tangier, 90000, Morocco; Orthopedics and Trauma Surgery Department, Mohammed VI University Hospital Center Tangier, Faculty of Medicine and Pharmacy, Abdelmalek Essaadi University, Tangier, 90000, Morocco; Orthopedics and Trauma Surgery Department, Mohammed VI University Hospital Center Tangier, Faculty of Medicine and Pharmacy, Abdelmalek Essaadi University, Tangier, 90000, Morocco; Orthopedics and Trauma Surgery Department, Mohammed VI University Hospital Center Tangier, Faculty of Medicine and Pharmacy, Abdelmalek Essaadi University, Tangier, 90000, Morocco; Orthopedics and Trauma Surgery Department, Mohammed VI University Hospital Center Tangier, Faculty of Medicine and Pharmacy, Abdelmalek Essaadi University, Tangier, 90000, Morocco; Orthopedics and Trauma Surgery Department, Mohammed VI University Hospital Center Tangier, Faculty of Medicine and Pharmacy, Abdelmalek Essaadi University, Tangier, 90000, Morocco

**Keywords:** pilomatricoma, elbow, adult, Benin

## Abstract

Pilomatricoma is a common benign skin tumor of follicular origin, occurring mainly in the head and neck region. However, less common locations, such as the arm or elbow, can also be observed. Definitive diagnosis is based on histopathological examination, and standard treatment consists of complete surgical excision of the lesion. We report the case of a 31-year-old man with a pilomatricoma located on the posterolateral aspect of the right elbow, which had been evolving for 2 years. After surgical excision under locoregional anesthesia followed by thin skin grafting, no signs of local recurrence were observed after 12 months' follow-up. This case is particularly interesting because of the rare location and large size of the tumor in the elbow.

## Introduction

Pilomatricoma is a common, benign skin tumor of the hair follicle, first described in 1880 by Malherbe and Chenantais. Previously known as Malherbe's calcifying epithelioma, it was renamed pilomatricoma by Forbis and Helwing in 1961. It is an adnexal tumor often overlooked and confused with other skin lesions. It occurs mainly in young subjects, under 20 years of age. The most common sites are the head and neck, while elbow involvement is exceptional [1, 2].

## Case report

**Figure 1:**
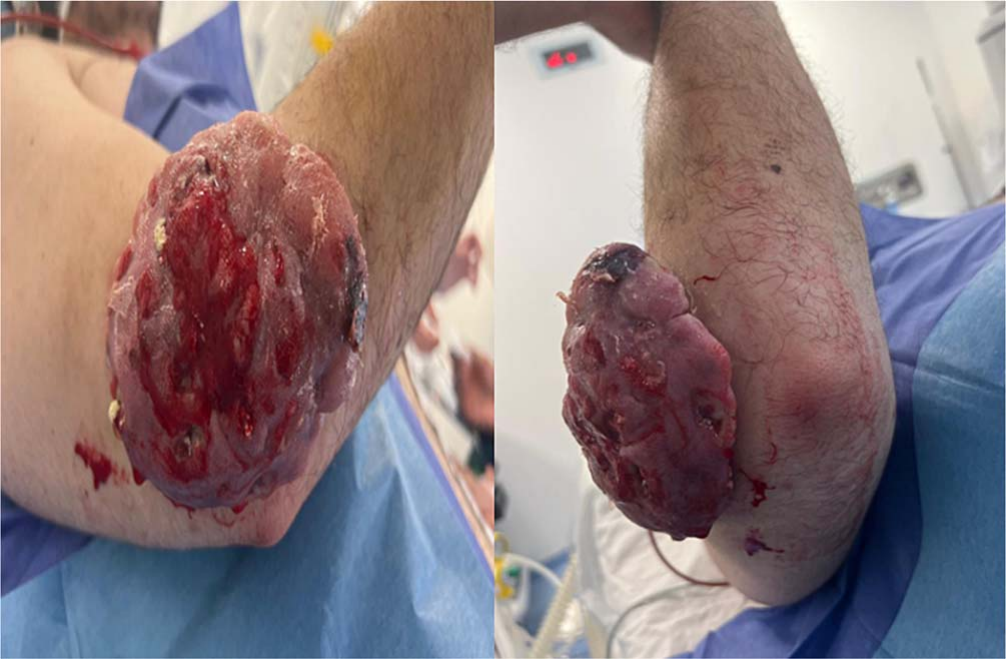
Clinical presentation of the patient.

The patient was a 31-year-old man with no previous pathological history of note, who presented with a painless swelling of the posterolateral aspect of the right elbow, which had been present for two years. The swelling was progressively increasing in size, with no signs of infection or bleeding. The patient was apyretic and in good general health. On clinical examination, he presented with a nodular mass on the posterolateral surface, ten centimeters in diameter, hard, painless, non-pulsatile, and adherent to the skin but mobile in relation to the deep plane. The skin was ulcerated and hemorrhagic. There were no downstream vascular or neurological disorders or satellite adenopathies, and elbow mobility was normal. Biological and inflammatory tests were normal (Fig. 1).

**Figure 2:**
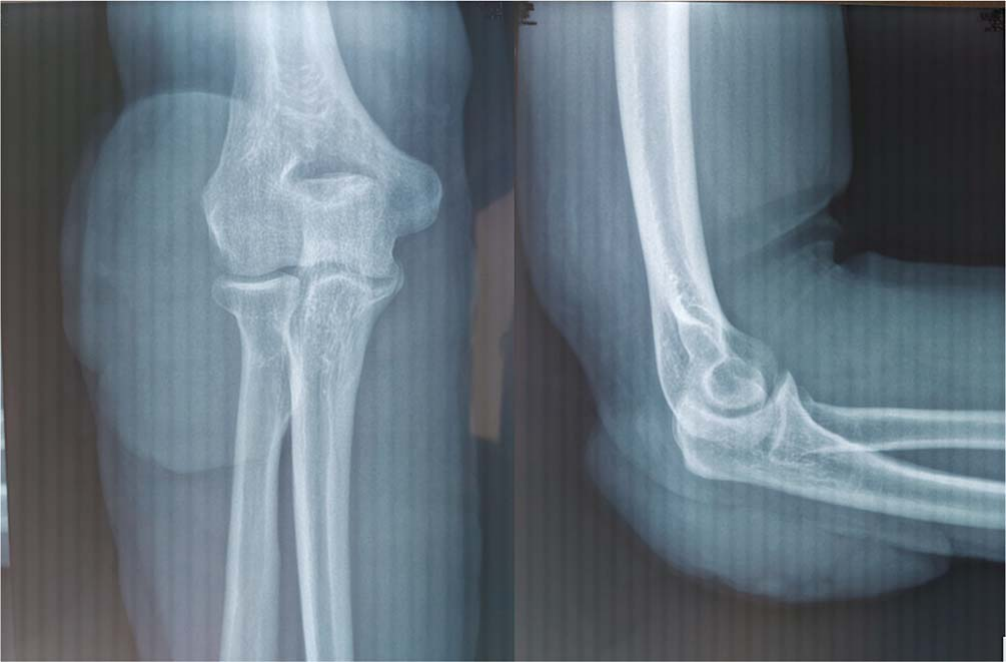
Front and side X-rays of the elbow.

The patient underwent a standard radiograph of the elbow, in front and in profile, showing no bone involvement and demonstrating the presence of a superficial extra-osseous oval opacity with a long vertical axis, with clean margins and no calcification, located posterolateral to the elbow. Surgical biopsy and histological study led to the diagnosis of pilomatricoma, with vasculoexudative inflammatory changes in the overlying skin tissue and no histological signs of malignancy (Fig. 2).

**Figure 3:**
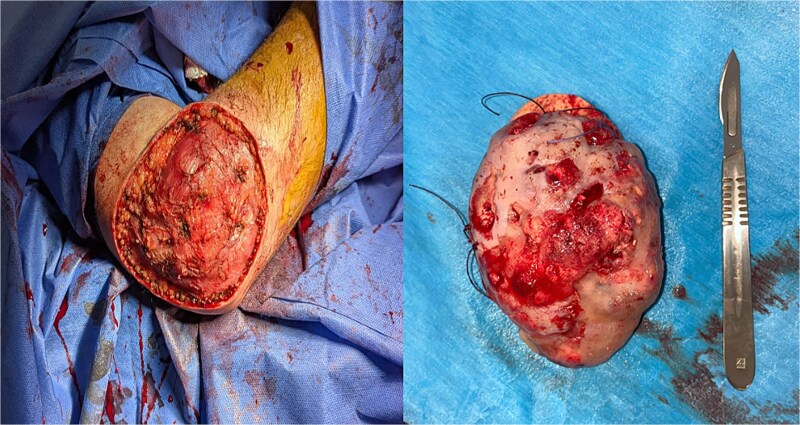
Surgical removal of pilomatricoma under general anesthesia.

Surgical excision under locoregional anesthesia, performed via a posterolateral approach, enabled total removal of this nodular, hemorrhagic, grayish-looking ulcerous tumor, with 2 cm safety margins. Pathological examination of the post-operative specimen revealed aggregates of regular basal-like epithelial cells, with mummification foci in the central zone, eosinophilic cytoplasm and hypocolorable nuclei, with clearly defined cytoplasmic outlines. With no signs of malignancy, the diagnosis of pilomatricoma was confirmed. The patient underwent a thin skin graft. Twelve months later, no signs of local recurrence were detected (Fig. 3).

## Discussion

 Pilomatricoma, or Malherbe's mummified epithelioma, was first described in 1880 by Malherbe and Chenantais as a benign, calcified tumor of the sebaceous glands [1]. Its origin was later confirmed by Forbis and Helwing, providing important validation for understanding the nature of this pathology [2].

This tumor can occur at any age, but is particularly common in children and young adults, peaking between the ages of 10 and 20. Several studies indicate that the majority of cases occur before the age of 20, which may be linked to the rapid proliferation of hair follicles and genetic factors favoring their formation. Our patient, aged 31, represents an atypical case [3].

There is a slight female predominance, although this difference is not always consistently observed in all studies. On average, 55%–65% of cases are reported in women. This female predominance may be influenced by various factors, including hormonal or genetic factors [4].

The tumor usually presents as a painless, often solitary subcutaneous mass, although multiple forms have also been reported. Its size can vary considerably, from a few millimeters to several centimeters [5]. Its preferred location is the neck and head, but cases have been described in the literature in other parts of the body, including the limbs, as in our patient [4].

Various clinical forms of pilomatricoma have been identified, in particular familial forms, often associated with systemic diseases such as myotonic dystrophy. In these cases, multiple localizations are frequent [6, 7].

Calcification of the tumor is present in around 80% of cases, sometimes forming a subcutaneous osteoma [2]. The usual size of pilomatricomas is less than three centimeters, although cases of giant pilomatricomas exceeding five centimeters in diameter have been reported. This is the case presented here [8].

The anetodermal form of pilomatricoma has been described, where the skin becomes flaccid due to compression by the tumor, resulting in the disappearance of elastic fibers [9]. This may lead to ulceration, complicating the differential diagnosis, particularly with cutaneous carcinoma. In our patient's case, the appearance of the skin was ulcerative-hemorrhagic, which posed a clinical diagnostic problem.

Clinical diagnosis is based on the appearance of the tumor, but it is often difficult to distinguish from other benign masses such as epidermal cysts or lipomas, making histopathological examination crucial. Ultrasound and computed tomography (CT) scans are sometimes used to better characterize the tumor and assess its extension [5]. However, recent studies show that clinical examinations and imaging have a low sensitivity for distinguishing pilomatricomas from other benign skin tumors [10].

On microscopic examination, pilomatricoma can be distinguished by the presence of basaloid cells arranged in a palisade around a central zone of necrosis, associated with keratinized cells and sometimes calcified deposits. Its well-defined, encapsulated appearance is a key sign. The use of hematoxylin–eosin staining confirms these characteristics and differentiates it from other benign lesions, such as epidermal cysts or lipomas, as well as from malignant tumors, such as basal cell carcinoma. Although clinical signs may be suggestive of pilomatricoma, only a histological diagnosis allows accurate identification and guarantees appropriate treatment [2–5].

Malignant transformation of pilomatricoma into carcinoma is an exceptional phenomenon, although a few cases have been reported, especially in recurrent or large pilomatricomas. However, this possibility is widely considered unlikely [3].

Treatment of pilomatricoma is essentially based on surgical excision, which remains the most effective method for avoiding recurrence and ensuring complete cure. Surgery is generally well tolerated and without major complications [11] although recurrence is rare, it can occur, particularly when the tumor has not been completely removed or in multiple forms of the pathology [4].

The prognosis for pilomatricoma is generally good. Cure without recurrence is the rule after total surgical excision [2–11].

Our case of pilomatricoma is remarkable for several atypical features, including onset in an adult, location in the elbow, significant tumor size (over 10 cm) and relatively rapid development. Several differential diagnoses have been considered in view of this clinical picture, with in particular the hypothesis of a malignant process, such as a trichomatricial carcinoma, known for its aggressive potential.

## Conclusion

Pilomatricoma is a rare benign tumor of the hair follicle, often localized on the head and neck, although cases on the limbs are exceptional. Diagnosis is based on clinical examination and histological confirmation. The main treatment remains surgical excision, leading to a complete cure in most cases. Although malignant transformation is extremely rare, post-treatment follow-up is essential to prevent recurrence.
